# Ternary network models for disturbed ecosystems

**DOI:** 10.1098/rsos.220619

**Published:** 2022-10-26

**Authors:** Kieran Peel, Darren Evans, Clive Emary

**Affiliations:** ^1^ School of Mathematics, Statistics and Physics, Newcastle University, Newcastle-upon-Tyne NE1 7RU, UK; ^2^ Natural and Environmental Sciences, Newcastle University, Newcastle-upon-Tyne NE1 7RU, UK

**Keywords:** biodiversity, cascading extinctions, population dynamics, network ecology, robustness

## Abstract

The complex network of interactions between species makes understanding the response of ecosystems to disturbances an enduring challenge. One commonplace way to deal with this complexity is to reduce the description of a species to a binary presence–absence variable. Though convenient, this limits the patterns of behaviours representable within such models. We address these shortcomings by considering discrete population models that expand species descriptions beyond the binary setting. Specifically, we focus on ternary (three-state) models which, alongside presence and absence, additionally permit species to become overabundant. We apply this ternary framework to the robustness analysis of model ecosystems and show that this expanded description permits the modelling of top-down extinction cascades emerging from consumer pressure or mesopredator release. Results therefore differ significantly from those seen in binary models, where such effects are absent. We also illustrate how this method opens up the modelling of ecosystem disturbances outside the scope of binary models, namely those in which species are externally raised to overabundance. Our method therefore has the potential to provide a richer description of ecosystem dynamics and their disturbances, while at the same time preserving the conceptual simplicity of familiar binary approaches.

## 1. Introduction

Anthropogenic drivers including climate change and habitat loss pose a severe threat to global biodiversity [1–5]. Current estimates warn that roughly a quarter of species in some animal and plant groups are threatened with extinction [6], and that one million species could go extinct soon unless urgent action is taken to reduce human impacts [6]. Understanding the knock-on effects of species loss in ecosystems and on the ecosystem services that they provide [7–10] represents, therefore, an urgent scientific challenge. Central to this endeavour is to account for the complex network of interactions within which each species is embedded. This is critical because network effects can set into motion cascades of secondary extinctions [11–13] and potentially lead to ecosystem collapse [14,15]. Recent work has highlighted the utility of network approaches to effective conservation strategies [16] and preservation of ecosystem services [17].

The response of ecological networks to species loss can be modelled with population dynamics techniques [18–27]. This gives detailed information about species abundances over time but suffers from being computationally time-consuming for large ecosystems and requiring a large number of, typically unknown, parameters to specify such a model. Qualitative approaches that capture the essential behaviours of complex systems therefore have a long history in ecology, e.g. [28–32]. One such alternative approach relevant here, with the advantages of simplicity and greater tractability, is to reduce the description of a species population to a single binary variable indicating its presence (+1) or absence (0) in the ecosystem in question. The composition of an ecosystem is then reduced to a binary string [33] and the dynamics of the ecosystem can be understood as an evolving Boolean network [34], most familiar in a biological context from the modelling of regulatory networks [35,36]. Such Boolean models have been fruitfully employed in the context of community assembly [37–40] where, despite their simplicity, they have been shown to replicate some important behaviours found in more complicated population dynamics models [39]. More relevant to this paper and its theme of ecosystem disturbance are models of community *disassembly*, where the widespread topological robustness approach to secondary extinctions [11,14,41–45] can be cast in this Boolean framework. In such models, primary extinctions are introduced either randomly [45] or according to a set of rules [14,45] by flipping the state of a species from 1 to 0, thus removing its participation in the network. A further set of rules then determines whether further nodes also then flip to 0 in response, indicating a secondary extinction. The robustness of the network is then assessed by the scale of secondary extinctions as a function of the number of primary extinctions.

Within this approach is disturbances of previously functional ecosystems can only ever give rise to ‘losers’ and never ‘winners’, i.e. species that thrive in their modified circumstance. This is unrealistic. For example, despite a dramatic overall decline in Lepidoptera species in the UK, 25% of macro-moth species have shown an increased abundance in the last five decades [46,47]. Similarly, 20–30% of wild bird species in the UK were observed to have increased in abundance compared with a background of overall population decline [48]. Furthermore, it is known that the network effects of these ‘winners’ can be vitally important in understanding the cascading effects of disturbances [49,50]. A classic example is the extinction of sea otters from the Pacific coasts of North America. This led to a dramatic increase in the abundance of their sea-urchin prey which in turn led to overgrazing of giant kelp and the subsequent collapse of formerly specious kelp forest communities [51]. This mesopredator release has been implicated in the extinction of many prey species [52,53].

The aim of this paper is to consider the expansion of discrete ecological network models from a binary description of species abundance to a *ternary*, i.e. three-state, one in which the additional state represents the species being significantly overabundant in the ecosystem. In this way, we seek to encompass the roles of both ‘winners’ as well as ‘losers’ in these discrete models. We begin by defining and motivating these ternary models, and then exploring some of their formal properties. We consider two test scenarios for the approach: the robustness of model ecological networks under sequences of species deletions and the effects of an initially positive perturbation on the same. In so doing, we highlight the differences in outcomes between binary and ternary approaches, and expand on the potential of the ternary approach for future modelling of ecosystem disturbances.

## 2. Ternary network models

In a ternary network model, the condition of a species is represented by one of three states, which we label −1, 0 and +1. State −1 corresponds to the (local) extinction of the species, comparable to the absent state in a binary approach. State 0 corresponds to abundance of the species being within the levels typically observed in the natural ecosystem in the absence of the disturbance in question (but with the presence of the usual complement of interactions between species in the ecosystem). We will refer to this state as the ‘equilibrium’ state, even though the interpretation need not be as a strict equilibrium—the state only need be assumed approximately stationary over the time scales relevant to the considered disturbance. Lastly, we introduce the third, new state +1 to represent the species having an abundance significantly in excess of the values associated with the 0 state. From a consumer–resource perspective, the abundances represented by the +1 state would be those comparable to the carrying capacity, i.e. freed from constraints imposed by significant interspecific interaction and limited therefore primarily by intraspecific interactions. This +1 state we refer to as the ‘overabundant’ state [54]. Compared with the binary approach, the ternary model splits the ‘present’ state into two states: equilibrium and overabundant. Naturally there is some arbitrariness in where to draw the distinction between these two states, but this is in part addressed by the introduction of a threshold parameter—see later.

Taking full account of the repercussions of these new states goes hand-in-hand with including top-down effects, typically absent in robustness calculations [13,44,55], such that an overabundant consumer (state +1) can negatively affect the survival chances of its prey.

**Figure 1.**
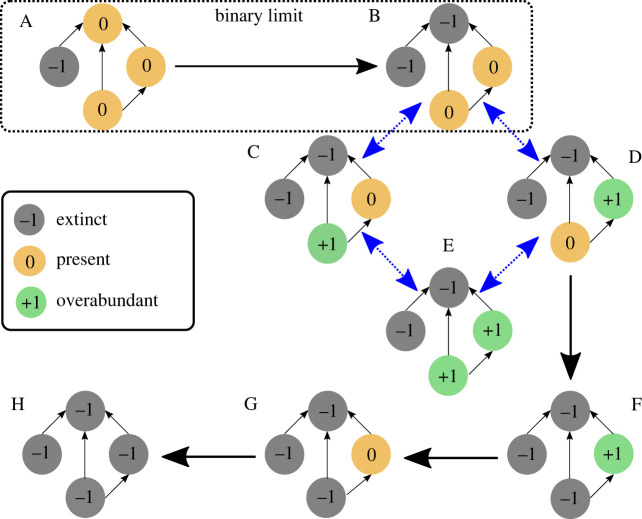
A comparison of the evolution of a four-species food web in binary and ternary models. Nodes represent species and are labelled as either present and largely undisturbed (0), extinct (−1) or overabundant (+1); edges represent trophic interactions directed from resource to consumer. Transitions between network states are driven by trophic interactions, with possibilities shown by thick arrows, either reversible (blue dashed) or irreversible (black solid). The starting point (A) consists of a single extinction (−1) in a network that is otherwise in equilibrium (0). In the transition A → B, the top predator has insufficient resources and becomes extinct. This transition represents the extent of the dynamics possible within a binary model. In contrast, a ternary model can show much richer behaviour. In the transitions B → C and B → D, the removal of top-down pressure causes prey species to become overly abundant. This has the potential to cause further transitions and could result in convergence to a loose attractor (BCDE if transition D → F is forbidden), or even complete ecosystem collapse, as in H.

The impact of this expanded description can be appreciated from figure 1, which shows possible transitions in a few-species food web. The starting network (A) consists of three species in equilibrium state 0 with a fourth species made recently extinct (−1). Since the extinct species is a resource species its consumer can, depending on the relative importance of the interaction and tolerance to extinction, become extinct as in (B). In a binary model, this would necessarily be the end of the story. However, with three states and top-down effects, further transitions are possible. In particular, removal of the apex predator releases consumer pressure on its remaining resources, allowing them to increase their abundances (C and D). This may in turn result in further transitions. For example, the top-down effect of increased predation driving a prey species to extinction is observed in the transition D → F. Possible end states for the binary and ternary models are thus very different: in the binary case, secondary extinction effects are limited; in the ternary case, disruption can spread much further through the network, even leading to complete ecosystem collapse (H). The increased state space of the ternary models also opens up new behaviours such as reversible transitions (blue dashed arrows in figure 1) as well as the emergence of *loose attractors* [56,57] in which the system remains indefinitely within a finite, typically quite small, subset of states (e.g. if transition D → F is forbidden, the set (BCDE) in figure 1 represents such an attractor).

## 3. Food-web robustness

To assess the impact of these effects in large networks, we consider the response of food webs to a range of disturbances. We take network dynamics to be governed by a discrete-time stochastic model that employs a ternary description of species states and includes top-down effects (see appendix A). A key parameter in this model is the threshold *ε* which governs the resistance to change of the species states. This is a generalization of what, in a topological knockout model [41], would be the typical proportion of prey species that a predator can lose before becoming extinct (see electronic supplementary material, text). The model also includes a parameter *d* that describes the strength of intra-specific competition relative to the trophic interactions of the food web. The combination of these two parameters dictates the type of attractor obtained in the model. Point attractors, i.e. attractors comprising a single, stable configuration, dominate where competition is small relative to the threshold, and here we focus on results obtained in this regime (see electronic supplementary material, text and figure S1 for more information on the relationship between parameters and attractor type).

**Figure 2.**
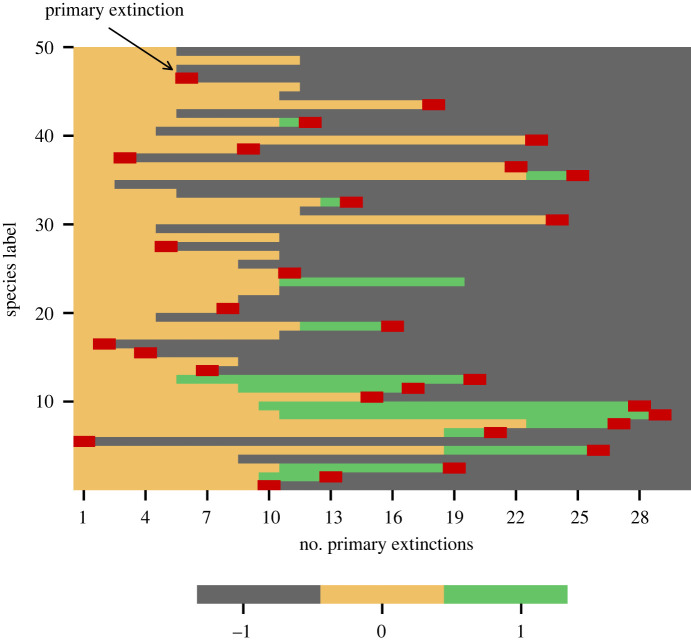
The network attractor found following each primary extinction in a single robustness sequence for a niche-model food web of *S* = 50 species. Species are ordered by their position in the food web with 10 basal species featuring at the bottom. Species states are colour coded according to their ternary state; red markers indicate the primary extinction that gave rise to the attractor displayed in that column. Large numbers of secondary extinction occur when the number of primary extinctions reaches 5, and again at 11. Following this second cascade, in particular, a significant number of species enter the +1 state, indicating that these states play a significant role in determining the robustness of such systems. The food web here had a connectance *c* = 0.1, threshold and competition parameters were *ε* = 0.5 and *d* = 0, respectively.

We begin by considering the robustness of food webs under sequences of primary extinctions [41,42,44,45]. In figure 2, we show the attracting configuration found after each primary extinction in a single robustness trajectory for a single instance of a niche-model food web. Species are ordered by their trophic position in the food web as determined by the metric designed by Levine [58]. For small numbers of primary extinctions, we see that secondary extinctions are rare. However, as the number of primary extinctions increases, significant cascades of secondary extinctions occur with many simultaneous transitions 0 → −1. Alongside the negative effects of the second cascade in particular, we also see several species move to the overabundant +1 state. These are particularly concentrated in lower trophic positions and after a large proportion of species have been removed, the remaining species tend to be basal species that have lost all predators and are thus fixed in the +1 state.

**Figure 3.**
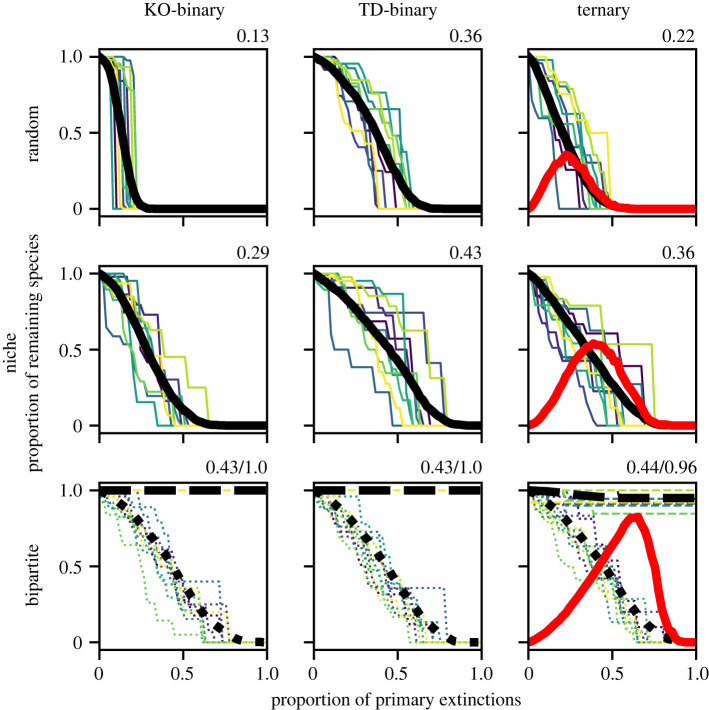
Robustness curves for food webs with random, niche and bipartite topologies calculated within knock out (KO) binary, top-down (TD) binary and ternary approaches. These curves show the fraction of species remaining following an increasing proportion of primary extinctions. Results for individual randomly generated topologies are shown with thin coloured lines, with the mean over 250 such curves shown in black. The numerical value next to each plot is the overall network robustness, i.e. the area under the mean curve. Across random and niche networks, the TD-binary model yields higher robustness than the KO model, but this is decreased in the ternary model. The red line for the ternary model indicates the proportion of remaining species in the attractor found in the +1 state—illustrating the role played by these states in decreasing robustness. For the bipartite network, curves are separated into predators (dotted) and prey (dashed) extinction curves, with the robustness of the predator curve given first. Here we see that only in the ternary model can prey species go extinct, and this because of downwards pressure of overabundant predators. Parameters as in figure 2.

Figure 3 shows robustness curves, i.e. the fraction of remaining species in the absorbing configuration as a function of the proportion of primary extinctions, for model food webs with three different topologies: (Erdos–Renyi) random, niche and bipartite. In each case, the order of the primary extinctions was chosen at random. Results of the ternary model are contrasted against those from two binary models, one with top-down (TD) binary and one without knockout (KO) binary top-down effects. Comparing the two binary models, we see that the inclusion of top-down effects significantly increases the robustness for random and niche networks. This is because, whereas removing a species from the middle of the food web depletes the resources of its consumers and thus makes their secondary extinction more likely, with top-down effects it also reduces pressure on the resources of the removed species, making their subsequent secondary extinction less likely. Moving from the TD-binary to ternary model for these networks, we see that the robustness decreases. This may be understood through the opening up of further pathways via +1 states to secondary extinction as in figure 1.

In the bipartite case, robustness results are shown for predator and prey species independently. While results for the KO and TD binary models differ for individual trajectories (due to the stochastic nature of the model), their aggregate behaviour is identical. This is because in binary bipartite models the only possible secondary extinctions are of predator species, for whom top-down effects are absent. In the ternary model, however, the secondary extinction of basal species also becomes possible due to the top-down effect of overabundant predators. This effect is nevertheless observed to be small here and the prey species remain significantly more robust than predator species.

The red lines in figure 3 show the proportion of remaining species found in the +1 state after the model has converged following a primary extinction. This gives an indication of the importance of these overabundant states in determining the dynamics of the system. As is clear, the rise in the prevalence of overabundant states coincides with where the fall in the robustness curve is most pronounced. This is where primary extinctions are causing the most serious disruption to the network—what is new here is that this disruption causes both extinctions and overabundances. While the specifics of the robustness curves and the location of the fall in the robustness curve depend strongly on the threshold, the qualitative differences between models persist (see electronic supplementary material, figure S2).

**Figure 4.**
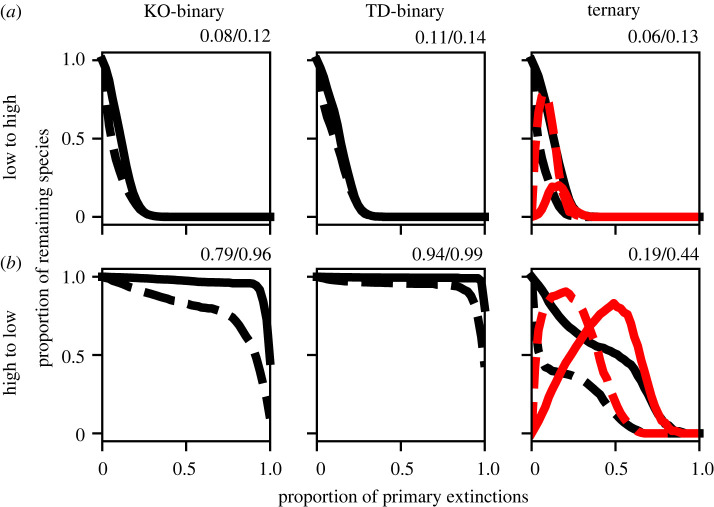
Robustness curves as in figure 3 for the niche model, but here for two non-random orderings of the primary extinctions sequence. In (*a*) the species removed at each step is that with the lowest position in the food web; in (*b*) it is the species with the highest position. Results are shown for two values of the threshold, *ε* = 0.5 (solid) as in figure 3 and *ε* = 0.25 (dashed). Robustness in the low-to-high scenario is strongly reduced relative to the random sequence and, as top-down effects are minimized by this sequence, all models give similar results. With the high-to-low sequence, we see that the KO- and TD-binary models show a large increase in robustness over the random sequence that is not reflected in the ternary model.

The bipartite models show that the trophic position of an extinction within a food web can affect the transitions, both positive or negative, that it will induce. To investigate this further, we consider two additional orderings of the primary extinction sequence: ‘low-to-high’, in which the next species removed is that closest to the bottom of the food web; and ‘high-to-low’, in which it is the topmost species. Robustness curves for these primary-extinction sequences in the niche model are shown in figure 4 (example attractor sequences are shown in electronic supplementary material, figure S3). In the low-to-high sequence, by removing the lowest-lying species first, we minimize the extent for which top-down effects can manifest. Correspondingly, we see that all three models give very similar results, with rapid collapses of the food webs as resources are removed. While the ternary results show that a fraction of species do enter the +1 state, this is smaller than with the random ordering, and has a correspondingly small effect on secondary extinctions. By contrast, removing the highest predators first has two main effects. Firstly, robustness increases in the two binary models. This is as expected, since resource species are preserved for longer in this sequence. The second effect is that with this ordering, the impact of top-down effects is exaggerated. This means that in the TD-binary model, the systematic removal of consumer pressure results in an extremely high robustness. In the ternary model, however, removal of consumer pressure leads to many overabundant states, the knock-on effects of which destabilize the network and produce a severe reduction in the robustness. The strength of this effect increases as the threshold parameter *ε* is reduced.

## 4. Positive disturbances

**Figure 5.**
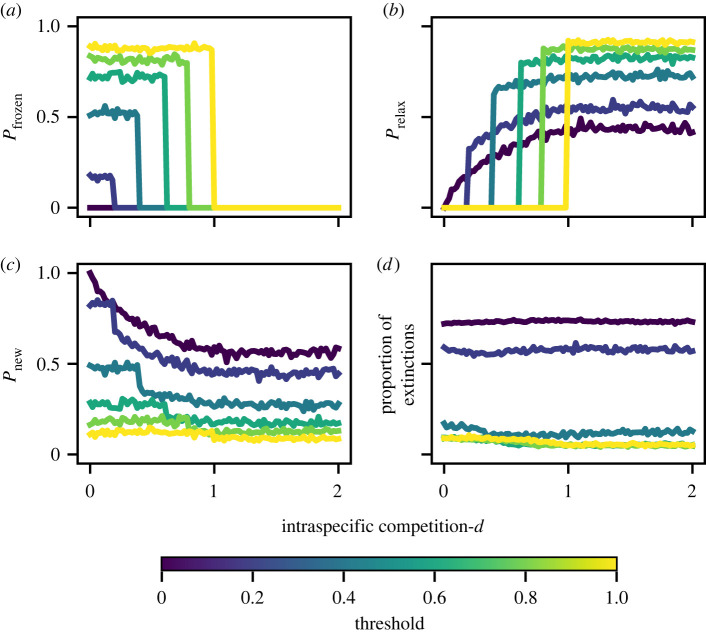
Outcomes of disturbing a single apex predator species so that it becomes overabundant (*z*_*i*_ = +1) in a niche-model food web of 50 species. The first three panels show the probability that (*a*) the +1 state simply remains frozen with no further effect, (*b*) the disturbed species relaxes back to its equilibrium state with no further effect, and (*c*) neither of these outcomes occurs and the network finds a new attractor. Panel (*d*) shows the mean proportion of extinctions in this new attractor. Results are plotted as a function of the intraspecific competition strength, *d*, and for a range of values of the threshold, *ε*.

The inclusion of the overabundant species in the ternary model enables the investigation of disturbances outside the scope of binary models. The consequences of promoting a single species to overabundance in a niche-model food web otherwise in equilibrium is explored in figure 5. Three classes of behaviour are observed: the overabundant state can freeze with no further effect such that the disturbed configuration is an attractor itself; the overabundant state can ‘relax’ back to its equilibrium value with no further effect; or, the overabundant species can induce transitions in its neighbours with knock-on effects that spread out through the network, giving rise to secondary extinctions. Figure 5*a*–*c* shows the probabilities of these three outcomes as a function of the intra-specific competition strength, *d*, and for different values of the transition threshold *ε*. We observe that when the competition strength is greater than the threshold, *d* > *ε*, relaxation dominates. Otherwise the behaviour typically freezes or moves to a new equilibrium, depending on whether the threshold is large or small. Figure 5*d* gives an indication of what happens when a new equilibrium is found. Here we plot the mean fraction of species extinctions in the new (i.e. non-frozen, non-relaxed) attractor. This quantity varies strongly as a function of the threshold, but is largely independent of the intra-specific competition. This is despite the fact that for *d* > 2*ε*, the dominant type of attractor switches from point to loose (see electronic supplementary material, text). The results here then indicate that the number of extinctions is similar in both point and loose attractors with similar parameters.

Figure 5 shows results obtained from disturbing an apex species. Analogous results for disturbing a basal species are given in electronic supplementary material, figure S4, and are found to be very similar. The small differences stem from how the species around the perturbation respond to it. The only effect a positive apex perturbation can have on its neighbours is to cause species extinction due to additional top-down pressure. This means that relaxation behaviour can only occur in the first timestep, since, if a secondary extinction is generated, the configuration no longer has the possibility to relax to the starting equilibrium. By contrast, a positive basal perturbation can cause its neighbours to move to the +1 state due to the increase in resources, and relaxation from this state is still possible even though the initial perturbation has spread to other species.

**Figure 6.**
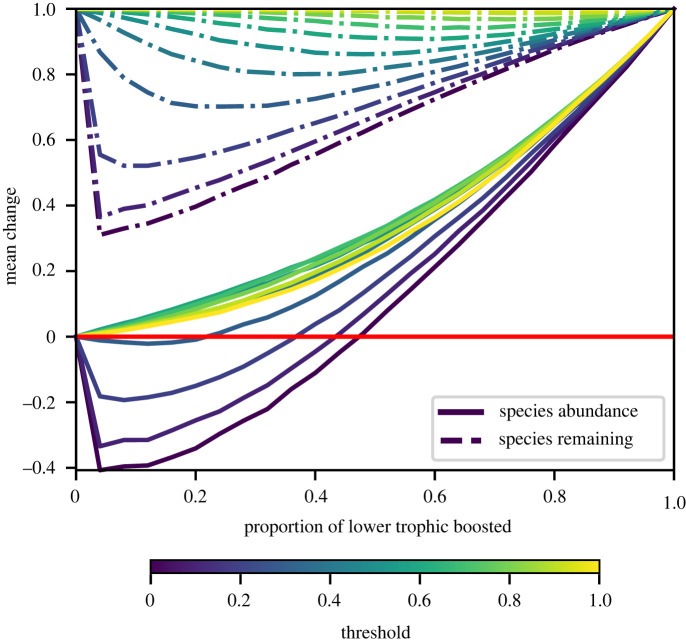
A proportion of the lower trophic level of a bipartite food web is boosted to the overabundant *z*_*i*_ = +1 state and held there. Here we plot the proportion of species remaining in the ensuing attractor, measured relative to the number of unfixed species, as well as the mean abundance change of these species. Results are given as a function of the fraction of boosted species and for a range of threshold values. When the threshold is high, we observe a positive gain in the mean abundance with few secondary extinctions. We set *d* = 0 here and the boosted species were chosen with the highest degree first. The networks consisted of *S* = 50 with a connectance of 0.1.

Finally, we consider a disturbance which boosts a subset of species to overabundance and holds them there. Such a scenario might pertain following a rapid change in external conditions that favours certain species, or as a result of management interventions looking to influence species abundances more broadly through the network. Specifically, we here consider a bipartite network structure, boost and hold a fraction of basal species, and we assess the effect of this disturbance with two metrics. The first is the fraction of non-boosted species that persist (i.e. state not *z*_*i*_ = −1), and the second is the mean abundance change of the unboosted species in the attractor (obtained by simply summing their *z*_*i*_ values). Figure 6 shows results obtained when the species boosted are the highest-degree basal species in the network. The outcome of this disturbance very much depends on the parameter regime. If the threshold is low, then for all but the smallest number of boosted states, we see significant species extinction and a corresponding reduction in mean abundance. On the other hand, if the threshold is high, extinctions are greatly suppressed and the overall change in the abundance is positive. For high threshold, in the region where 15–20% of basal species are fixed, the mean abundance change is positive and the fraction of species remaining is close to one. In the regime then, where species are individually robust to changes in their local network environment, it is possible to manipulate the ecosystem to yield a knock-on increase in the abundances of other species.

Results where the boosted species are chosen randomly or by having the lowest degree are shown in electronic supplementary material, figure S5. If our goal is to increase abundance while minimizing species loss, then the strategy of boosting the most highly connected basal nodes first, i.e. the strategy employed in figure 6, is the most successful. This mirrors findings in binary approaches which have shown that removal of least-connected species first induces the fewest secondary extinctions [42].

## 5. Discussion

We have described an approach to the modelling of ecosystem disturbances that expands the widespread binary approaches to include the notion that species can gain as well as lose from the disturbance. Alongside this, we have included top-down effects such that overabundant consumers act deleteriously on their resources. We have seen that the inclusion of these effects can drastically modify the results of robustness analyses: the inclusion of top-down effects generally increases robustness; the inclusion of overabundant states reduces it. How these two effects combine depends very much on the specifics of the scenario under investigation, and consequently, binary model results can either under- or over-estimate extinctions of the full ternary calculation. We found that the ordering of the sequence of primary extinctions in terms of their position in the food web has a large effect on how this comparison plays out. Top-down and overabundance effects were most pronounced when species were removed from the top of the food web first. This would be especially pertinent in modelling aquatic food webs subject to ‘fishing down the food web’ where fishing preferentially targets larger, higher trophic-level species but, as a result of collapsing stocks, moves down trophic levels to keep up with demand [59,60]. We find that niche-model food webs are more robust than random ones across both binary and ternary approaches. On average the highest levels of robustness are observed in bipartite networks where, despite secondary extinction of basal species being possible in the ternary model, this happens very infrequently due to a lack of sufficient top-down pressure. More than just modifying the behaviour of previous approaches, the ternary structure also opens up the study of new ecosystem disturbances, ones obviously out of the scope of binary models. These are disturbances in which species are externally driven to overabundance, such as could occur through sudden changes in the growth conditions, or through management intervention. Invasive species might be fruitfully modelled in this framework because, following introduction, the superior traits of the invader can give an initial rapid growth [61,62] such that an overabundant disturbance might be the appropriate starting point for studying displacement of native species. As response to this type of positive perturbation, the ternary model displays a mixture of behaviours from trivial freezing and relaxation to extinction cascades and attractors in which boosted basal species stably support an increasing abundance in consumer species.

Moving to a ternary structure enables the formation of loose attractors in closed systems, i.e. without immigration. As described in the electronic supplementary material, text, we expect loose attractors to become prevalent in our model when the intraspecific competition satisfies *d* > 2*ε* (see also electronic supplementary material, figure S1). Otherwise point attractors dominate. Electronic supplementary material, figures S6 and S7 show the changes to our robustness analyses that result from using a value of *d* = 1 instead of *d* = 0 (as in figures 2 and 3). Despite loose attractors dominating in this parameter regime, these results resemble closely those obtained with *d* = 0 and point attractors. A similar comparison can be made between figures 6 and electronic supplementary material, S8 for a positive perturbation. Thus, we conclude, although the dynamics leading to this final state can be very different, the final number of extinctions is relatively insensitive to whether we have point or loose attractors.

One interesting aspect of the dynamics of loose attractors is that they can show apparent interactions between species. Consider two species with states (*z*_*i*_, *z*_*j*_) and no direct interaction between them. If in the loose attractor there appears states (0, + 1) and ( + 1, 0) but without ( + 1, + 1) (or at least with a frequency significantly reduced compared with what one would expect if the two 0 ↔ 1 transitions were independent), then the conclusion would be that there is an apparent competition between the two species brought about by the effect of the rest of the network. Binary models have the scope to show something similar but for the states (0, − 1) and ( − 1, 0), which involves extinction and re-invasion. In the ternary approach, we can see this effect when both species are permanently present.

We have chosen here to employ a stochastic model of the dynamics within this framework. This approach therefore has some similarities with the stochastic ecological network occupancy (SENO) model [40] with its discrete states, stochastic transitions and the inclusion of top-down effects. However the SENO model is a binary presence–absence model primarily aimed at understanding community assembly. In it, top-down effects were incorporated as extirpation rates of prey species, set against a competing tendency for recolonization through immigration. By contrast, the work described here is based around a starting equilibrium state with top-down effects incorporated as part of overall transition rates, which can then be either positive or negative depending on the direction of deviation from equilibrium. The ternary model uses three states as the minimal modification to binary/presence–absence approaches that enable treatment top-down effects. Moreover, the change from a binary to a ternary description signifies a qualitative change in the possible behaviours in state space for a closed ecosystem, i.e. one without re-invasion, in that reversible transitions become allowed and with them loose attractors. Nevertheless, one could consider the possibility of adding more discrete states, with the reasoning being that the more states, the closer continuous population-dynamics might be approached. Of the additional levels that could be added, one that represents a significantly diminished but not yet extinct population has the greatest potential to add significantly to the dynamics. This is because it gives state space enough to allow species to recover from negative perturbations. Going further, we can imagine a mapping between the discrete states in a multi-state model and the IUCN red list categories [63,64], hence providing a connection between this disturbance model and real-world threat assessment. This added level of detail would come at a price, however. For a system of *S* species, each of which is described by *L* discrete states, the number of possible configurations is *L*^*S*^. Thus, even the move from *L* = 3 to *L* = 4 brings with it a significant computational cost for large networks. In this context, we note that the results presented here were obtained using an asynchronous update rule, in which at most one species changes state during each timestep. Moving to synchronous updating [37,65], where multiple species changes are possible per timestep, offers a way to speed up calculations. Although this change would affect the details of the dynamics, the attracting configurations would remain the same.

Even staying within the ternary framework, significant avenues for improvement and future work exist. The transition rates in the model are determined by a ‘condition function’ that describes the total pressures on a given species (see appendix A). We have here taken this function to have a linear dependence on *z*_*i*_. This is illustrative and it remains a future question whether nonlinear functions may provide better models of ecosystem disturbances [13]. The ternary approach seems particularly well suited to mutualistic systems, where mutually beneficial interactions in populations dynamics naturally lead to overabundance if other factors are not taken into account [66]. Such interactions are easily incorporated into the condition function and thus ternary approach is readily adaptable to other networks, interaction types and functional forms.

We conclude by noting that, although we have provided logic and justification for studying ternary models and have shown that they can exhibit behaviours that differ significantly from those seen in binary models, ultimately it is the ability of a model to describe patterns in nature that will be the judge of its utility. A first step in making the connection with real ecosystems is to use empirically observed networks instead of the generic model ones used here. In the case where network edge weights are known, these can be used as proxies for the interaction coefficients in the condition functions. This still leaves undetermined important model parameters, in particular the threshold *ε* and the strength of top-down effects *β*, which would have to be taken as parameters to be determined. Predictions of such models could then be compared with the species abundances arising in disturbed ecosystems. An analysis of this sort has been conducted for binary models [67], in which the disturbance was the experimental removal of a sequence of plants from real plant–pollinator networks. Comparison of the observed behaviour with predictions from various binary models indicated that extinctions were lower in the models than were observed in experiment and that generally the networks reacted in ways different to those expected from the binary models. Whether ternary models could address these failings remains to be seen. Empirical networks in which apex predators have been suppressed could also be a scenario where ternary-model dynamics could be well tested. For example, one study investigated suppressing dingo populations, which found increased abundances in direct competitors and prey, but fewer rodents, indicating ‘winners’ and ‘losers’ that arise from such a disturbance [68]. Conversely, networks where predators have been reintroduced could demonstrate how the restoration of top-down control impacts prey populations [69]. In any case, direct comparison with experiments is certainly a challenge. A potentially useful intermediate step would be to compare the results of binary and ternary models with more detailed simulations of ecosystems dynamics, such as consumer–resource models [70], potentially subject to noise [71]. In this way, we might generally establish the conditions under which we expect the ternary approach to provide improvement over binary ones. This line of research would also establish whether discrete-state models such as those considered here should be treated simply as heuristic models of the full dynamics or whether they can be obtained as effective models from more microscopic ones by, e.g. a course-graining process.

## Data Availability

Data supporting the results are available from the Figshare repository at http://doi.org/10.25405/data.ncl.16855699 [72]. The data are provided in electronic supplementary material [73].

## Appendix A. Stochastic ternary network models

We describe the state of species *i* by the ternary variable *z*_*i*_ ∈ { − 1, 0, 1} corresponding to ‘extinct’, ‘close to equilibrium’ and ‘overabundant’. The total state of the network is hence given by the configuration $z\;z=(z_{1},\ldots z_{S})$, with *S* the number of species. Time evolution is taken to be discrete, stochastic, and homogeneous in time. We denote as $P_{i}^{\pm }(z\;z)$ the probability that transition $\left(z_{1},\ldots ,z_{S})\to (z_{1},\ldots z_{i}\pm 1,\ldots ,z_{S}\right)$ occurs within a given timestep. The dependence of probabilities $P_{i}^{\pm }$ on the species configuration zz is determined by the interactions of the species with the other species in the ecological network. We take the updating of the network configuration to be asynchronous, therefore at most one species can change state in each timestep, such that the set of probabilities is complete, $\sum_{i=1}^{S}\sum_{s=\pm }P_{i}^{s}(z\;z)=1$. For the disturbance scenarios studied here, we assume that the configuration zz=00 is stable, which implies $P_{i}^{s}(0\;0)=0$, for all *i* and *s*. Dynamics are then driven by perturbations to this configuration.

We write the transition probabilities as $P_{i}^{\pm }(z\;z)={\tilde{P}}_{i}^{\pm }(z\;z)/\sum_{k=1}^{S}\sum_{s=\pm }{\tilde{P}}_{k}^{s}(z\;z)$ where, to remain within the configuration space we must have ${\tilde{P}}_{i}^{+}=0$ when *z*_*i*_ = +1 and ${\tilde{P}}_{i}^{\pm }=0$ when *z*_*i*_ = −1. The latter prevents extinct species re-establishing themselves.

We then further specify interactions to those of a food web. If all prey of species *i* are extinct then, providing it is not extinct itself, we have ${\tilde{P}}_{i}^{-}=1$ and ${\tilde{P}}_{i}^{+}=0$, such that species *i* will die out. In all other cases, we write

$${\tilde{P}}_{i}^{\pm }=|g_{i}|\;\theta (\pm g_{i}-\epsilon ),$$

where *θ* is the unit-step function, *ε* is a threshold for whether transitions take place or not, and *g*_*i*_ represents the ‘condition’ of species *i*, which for simplicity we take to be the linear function

$$g_{i}(z\;z)=\frac{\sum_{\;j=1}^{S}f_{ij}z_{j}}{\sum_{\;j=1}^{S}f_{ij}}-\beta_{i}\frac{\sum_{\;j=1}^{S}e_{ij}z_{j}}{\sum_{\;j=1}^{S}e_{ij}}-d_{i}z_{i}.$$

Here, *f*_*ij*_ and *e*_*ij*_ are edge weights that represent interaction strengths with *f*_*ij*_ > 0 and *e*_*ji*_ > 0 if species *i* preys on species *j*. The first term therefore represents the total influence of prey species on species *i* (bottom-up effects), and the second term represents the total influence of predators (top-down effects). Parameter *β* weights the relative importance of these two contributions. We have used a value of *β* = 1 for the ternary model results here. The final term in equation (A 2) represents the limiting effect of intraspecific competition with a strength *d*_*i*_.

**A.1. Binary models**

The knockout (KO) and top-down (TD) binary models can be obtained as modifications to the above. In both cases, the state of species *i* is restricted to set of states *z*_*i*_ ∈ { − 1, 0}. In the KO-binary model, top-down effects are additionally removed by setting *β* = 0. In both cases, the competition term ceases to be relevant, as it only contributes when *z*_*i*_ = +1.

**A.2. Simulations**

Three different random network structures were studied: the Erdos–Renyi model as baseline for random networks; the niche model as a simple model that captures many important properties of empirical food webs [74]; and a bipartite model with its clear division into predator and prey species. See electronic supplementary material, text for full information on the generation of network topologies and interaction strengths.

The results shown here are the average of 250 runs for the robustness curves and 1000 runs for the positive disturbance results. In each run, a different network topology and set of interaction strengths were generated. In the robustness results, where the three models (KO/TD binary, ternary) are compared, the same 250 topologies were used in each model to ensure the most direct comparison. In each run, time-stepping is performed until an attractor is reached, for which either all probabilities $P_{i}^{\pm }$ are zero or they are such that no more species can become extinct. This latter condition was assessed numerically and we found that iterating 10^4^ timesteps since the last observed extinction is sufficient that the loose attractor is converged with reasonable certainty (see electronic supplementary material, text and figure S1).
